# Role of alanine aminotransferase in crop resilience to climate change: a critical review

**DOI:** 10.1007/s12298-024-01540-8

**Published:** 2024-12-11

**Authors:** Nisha Agrawal, Rati S. Chunletia, Anand M. Badigannavar, Suvendu Mondal

**Affiliations:** 1 Nuclear Agriculture and Biotechnology Division, Bhabha Atomic Research Centre, Mumbai, 400085 India; 2 Homi Bhabha National Institute, Training School Complex, Anushaktinagar, Mumbai, 400094 India

**Keywords:** Biotic resistance, Climate change, Enhancer insertion, Genome editing, Hypoxia, Nitrogen use efficiency, Pre-harvest sprouting

## Abstract

Alanine aminotransferase (AlaAT) is a crucial enzyme present in various isoforms. It is playing vital role in both humans and plants. This concise review focuses on the role of AlaAT in plants, particularly on preharvest sprouting, hypoxia, nitrogen use efficiency, abiotic and biotic stress tolerance. The molecular genetics of AlaAT, including gene identification and the impact on plant yield and its physiology, is discussed. Notably, the major dormancy gene *Qsd1/SD1* governing AlaAT synthesis has been characterized and cloned in various crops. This review emphasizes the current understanding of AlaAT and its influence on plants, covering mechanisms regulating preharvest sprouting, hypoxia, drought tolerance, salt tolerance, biotic resistance and nitrogen use efficiency. Identifying a protein with multidimensional roles in crop plants is very important. Modern biotechnological approaches can alter such candidate gene/protein for superior varieties development. Overall, the review gives an understanding of the uncovered area of AlaAT and the challenge of climatic change triggers in plants. In the future, the potential of genome editing in AlaAT through enhancer insertion and rapid stabilization through speed breeding will impart resilience to crop plants against climate change.

## Introduction

Alanine aminotransferase (AlaAT; L-alanine: 2-oxoglutarate aminotransferase; EC 2.6.1.2) is an enzyme that catalyses the reversible transamination of an amino group from glutamate to pyruvate to form 2-oxoglutarate and L-alanine (Welch 1975). The enzyme catalyzes the reversible transamination reaction between alanine and 2-oxoglutarate according to the Ping Pong Bi-Bi mechanism (Raychaudhuri 2015). In comparison, β-alanine aminotransferase (EC 2.6.1.18) forms β-alanine (D-alanine) from a pydridoxal-5-phosphate (PLP) dependent transamination reaction from L-alanine and malonate semialdehyde (Hayaishi et al. 1961; Stinson and Spencer 1969a&b). Initially, it was identified in a sub cellular fraction from bean cotyledons (Stinson and Spencer 1969a). Alpha alanine or L-alanine is proteinogenic in nature while β-alanine is non- proteinogenic.

AlaAT is widely present in various plant tissues and organs. Many researchers documented that AlaAT plays an important role in hypoxia conditions by conserving carbon and nitrogen source in the form of alanine in *Hordeum vulgare*, *Medicago truncatula*, *Arabidopsis thaliana* and *Lotus japonica* (Good and Crosby 1989; Ricoult et al. 2005, 2006; Miyashita et al. 2007; Rocha et al. 2010). Splittstoesser et al. (1976) showed that AlaAT plays a key role in synthesizing alanine during germination in pumpkin cotyledons. Very recently, AlaAT activity was noticed in nectar, nectaries, and leaves of 30 *Pitcairnia* species and the highest concentration of alanine was found in nectar of reddish flowers (Göttlinger and Lohaus 2024). Studies on mammalian cells revealed that AlaAT is crucial in gluconeogenesis and amino acid metabolism and this familiar enzyme is used as a marker of liver function (Felig 1973). The ubiquitous presence of AlaAT in lower organisms (including archaea) to higher organism suggests that it is an ancient and highly conserved biomolecule (Sakuraba et al. 2004). AlaAT was initially isolated and purified from cotton seeds by Turano et al (1966) and later identified in various plant parts, including *Atriplex spongiosa* leaves, tomato fruit, and pumpkin cotyledons. This wide distribution across plant tissues suggests its vital role in plant life cycle. Plant-specific isozyme variations resulted in the detection of two to six isoforms of AlaAT enzyme located in different cellular compartments such as cytoplasm, mitochondria, and peroxisomes (Liepman and Olsen 2004). Wightman and Forest (1978) found similarities between plant and animal AlaAT in terms of molecular weight, sedimentation coefficient, subunit composition, pyridoxal phosphate requirement, pH and cation effects. While aminotransferase activity is abundant in the cytosol, there are suggestions that α-alanine aminotransferase might predominantly reside in mitochondria, facilitating the utilization of pyruvate produced through the Krebs cycle in plants (Bone and Fowden 1960; Yu and Spencer 1970). Apart from cytosol and mitochondria, this enzyme is also presents in peroxisome and plays a role in photorespiration (Liepman and Olsen 2003). In the present review, molecular understanding of this enzyme was highlighted initially. Later, we emphasize its importance in providing tolerance against pre-harvest sprouting hypoxia tolerance, nitrogen use efficiency, abiotic stress (drought, salinity and heat) tolerance and biotic stress tolerance in crop plants. There are several unique functions of β-alanine in plants. It is accumulated as a generic stress response molecule involved in protecting plants from temperature extremes, hypoxia, drought, heavy metal shock, and some biotic stresses (Parthasarathy et al. 2019). A novel cytosolic enzyme from *Phaseolus vulgaris* cotyledons showed that β-alanine could be converted into plant signalling molecule ethylene via the formation of malonate semialdehyde and/or β-hydroxypropionate (Stinson and Spencer 1969a, b). Moreover, involvement of β-alanine aminotransferases in drought and salinity stress response is also elaborated here. A detailed account of utilizing allelic variation or creating such variation through modern biotechnological tools is depicted for better use in agriculture.

## Basic understanding of alanine aminotransferase

### Molecular details of alanine aminotransferase

Wightman and Forest (1978) found that the aminotransferases or transaminases are pyridoxal-5′-phosphate dependent enzymes that catalyse reversible reactions between amino acids and α-keto (2-oxo) acids. Rech and Crouzet (1974) isolated AlaAT from tomato fruits for the first time. The Michaelis constants for alanine, 2-oxoglutarate, pyruvate, and glutamate were found to be 2.8 mM, 0.28 mM, 0.09 mM, and 2.3 mM, respectively. Good and Muench (1992) isolated the AlaAT enzyme from barley roots and found that molecular masses of the homodimer (native) and monomer are 97 kDa and 50 kDa, respectively. While, Son and Sugiyama (1992) characterized three isoforms of AlaAT from prosomillet (*Panicum miliaceum*) and found that it had 44.2–44.8% homology with rat and human liver AlaAT. The isoform AlaAT-2 is induced by light and function in the C4 cycle aspartate/alanine shuttle. Thereafter, Kikuchi et al. (1999) firstly isolated cDNA encoding plant AlaAT from rice and the primary structure of the enzyme was deduced with the nucleotide sequence of gene composed of 14 introns ranging from 66 to 1547 bp in length and has15 exons. Moreover, it was found that the rice cDNA was about 1.8 kb long, with an open reading frame of 1449 bp (483 amino acids), a 5’-UTR of 68 bp and a 3’-UTR of 280 bp. The deduced molecular mass of the protein was 52,590 Da, aligning closely with the 52 kDa determined by SDS-PAGE, which corresponds to an amino acid sequence of 482 residues (Muench and Good 1994). When the deduced amino acid sequences of AlaAT from rice, prosomillet (Son and Sugiyama 1992) and barley (Muench and Good 1994) were compared, the AlaAT from rice was found to be with 91% and 88% homology to AlaAT of prosomillet and barley, respectively. Duff et al. (2012) first crystallized plant-based AlaAT (from barley) in complex with PLP and L-cycloserine, resolving up to 2.7 Å. Their findings suggested a canonical aminotransferase fold similar to structures of *Thermotoga maritima*, *Pyrococcus furiosus*, and human. Cycloserine is a structural analog of alanine and is known to be a more effective inhibitor of AlaAT activity than other transaminases. The major activity for this crystallized enzyme was alanine aminotransferase rather than aspartate aminotransferase with a native homodimer (105,761 Da) comprised of two identical 53 kDa subunits.

*AlaAT* gene expression is regulated by various environmental factors like hypoxia (Diab and Limami 2016), temperature (Yamakawa and Hakata 2010), drought (Kendziorek et al. 2012) and biotic stress (Sempruch et al. (2012). Plants have different homologs of alanine aminotransferases. Apart from cytosolic and mitochondrial, few homologs are peroxisomal too. Three Arabidopsis homologs of alanine (Ala): glyoxylate aminotransferase 2 (AGT2) contain a putative type 1 peroxisomal targeting signal and participate in photorespiration (Liepman and Olsen 2003). Localization of mitochondrial AlaAT depends on the N-terminal targeting sequence for the transport of protein to the organelle (Metón et al. 2004). In addition to gene expression, protein activity is controlled through post-translational modification (PTM) that creates a fine-tuning between all regulatory networks (Pandey and Gayen 2024). PTM of aminotransferase is not an exception. Lysine methylation of aspartate aminotransferase from *Sulfolobus solfaturicus* was evident for the involvement in protein aging and turn over (Zappacosta et al. 1994). Through an in vitro experiment, Beránek et al. (2001) showed inhibition of AlaAT (from porcine heart) activity due to glycation with D-ribose, D-fructose and D, L glyceraldehyde. It was interesting to note that D, L-glyceraldehyde, an intermediate of glucose metabolism, was the most potent AlaAT inhibitor due to glycation at ε-amino group of Lys313 of the protein. Protein per-sulfidation is another way to control the activity of enzymes in response to various stresses in plants (Moselar et al. 2024). García-Calderón et al. (2023) and Jurado-Flores et al. (2023) first documented the per-sulfidation of alanine-glyoxylate aminotransferase (At4g39660), alanine aminotransferase 1 (At1g17290) and alanine aminotransferase 2 (At1g72330) in mitochondria of Arabidopsis.

### Isozymes of alanine amino transferase

Apart from its different homologs across plant species, each plant has different isoforms along its various tissues that have different function during development and exposure to environmental stresses (Table 1). In *Arabidopsis thaliana* genome, four genes that encode alanine aminotransferases are present (Igarashi et al. 2003). Of them, two alanine aminotransferases *AlaAT1* and *AlaAT2* were identified (NCBI accession: AAF82782 and AAF82781, respectively) and characterized. In silico prediction indicated that AlaAT1 is located in cytosol and AlaAT2 in mitochondria (Liepman and Olsen 2003). While GGAT1 and GGAT2 were the homologues of glutamate: glyoxylate aminotransferase. Besides, three alanine (Ala): glyoxylate aminotransferase 2 (AGT2) was found in Arabidopsis and these proteins contain a putative type 1 peroxisomal targeting signal (Liepman and Olsen 2003). Like other pyridoxal enzymes, AlaAT forms an aldimine bond with pyridoxal 5-phosphate (PLP), involving conserved lysine residues. Crucial amino acid residues for hydrogen bonding with PLP, including lysine residue 300, are conserved also in rice AlaAT, facilitating aldimine bond formation with PLP. Wiśniewski et al. (2006) estimated the molecular weights of AlaAT homologs from Arabidopsis to be approximately 61.8 and 62.9 kDa, and for GGATs, around 54.7 and 55.3 kDa. Kendziorek et al. (2012) isolated the four homologs of alanine aminotransferase, namely two copies of alanine aminotransferase (AlaAT 1 and AlaAT 2) and two copies L-glutamate: glyoxylate aminotransferase (GGAT1and GGAT2) in wheat too. AlaAT1 and AlaAT2 were found to have a molecular weight of 65 kDa in their native state. Utilizing alanine as a substrate, comparable kinetic constants for K_m_ and catalytic efficiency (k_cat_/K_m_) for L-alanine with 2-oxoglutarate were observed, indicating similarity in kinetic properties between AlaAT1 and AlaAT2. Furthermore, Duff et al. (2012) found that the barley AlaAT catalyzes the forward (alanine-forming) reaction with a kcat value of 25.6 s^−1^, the reverse reaction with a kcat value of 12.1 s^−1^, and has an equilibrium constant of 0.5. Xu et al. (2017) studied the *Populus trichocarpa* and identified four alanine aminotransferase homologs, classified into two subgroups, A and B. Out of them, AlaAT1 and AlaAT2 in subgroup B encode GGAT, whereas AlaAT3 and AlaAT4 in subgroup A encode AlaAT. They cloned four *AlaAT* genes from *P. simonii* × *P. nigra* in which *PnAlaAT1* and *PnAlaAT2* were expressed primarily in leaves and inhibited in roots. *PnAlaAT3* and *PnAlaAT4* were mainly expressed in roots, stems and leaves, and induced by exogenous nitrogen. During a proteomics study by Czernicka et al. (2022), it was observed that the gene *Solyc03g123610* is responsible for the alanine aminotransferase activity in hypoxia condition in roots of tomato plants.

**Table 1:** Involvement of AlaAT enzyme in various functions of the crops

Crops/plants	QTL/Gene	Functions	Organ	References
Barley (*Hordeum vulgare*)	*Qsd1/SD1*	Seed dormancy	Seed	Sato et al. (2016)
Wheat (*Triticum aestivum*)	*Qsd1*	Seed dormancy	Seed	Onishi et al. (2017)
Soybean (*Glycine max*.)	–	Hypoxia	Root	De Souse and Sodek ((2003))
Barrel clover (*Medicago trancatula)*	–	Anoxia stress during germination and seedling	Seed	Ricoult et al. (2005)
Rice (*Oryza sativa*)	*HvAlaAT*	Improve nitrogen use efficiency	Root	Tiong et al. (2021)
Wheat (*T. aestivum*)	*OsAnt1:HvAlaAT*	Improve nitrogen use efficiency	Root	Tiong et al. (2021)
Barley (*H. vulgare*)	*OsAnt1:HvAlaAT*	Improve nitrogen use efficiency	Root	Tiong et al. (2021)
Wheat (*T. aestivum*)	*Qphs.ocs-3A.1*	Grain dormancy	Seed	Mori et al. 2005; Nakamura et al. (2011)
Wheat (*T. aestivum*)	*AlaAT1* and *AlaAT2*	Nitrogen availability	Seedlings	Kendziorek et al. (2012)
Barley (*H. vulgare*)	*AlaAT*	Hypoxia	Root	Good and Crosby (1989); Good and Muench (1992)
Proso millet (*Panicum miliaceum*)	–	Hypoxia	Root	Muench and Good (1994)
Arabidopsis (*Arabidopsis thaliana*)	*AlaAT1*	Hypoxia	Root	Klok et al. (2002); Loreti et al. (2005); Miyashita et al. (2007)
Pumpkin (*Cucurbita moschata*)	*AlaAT*	Germination	Pumpkin cotyledons	Splittstoesser et al. (1976)
Lotus (*Lotus japonicas*)	*AlaAT*	Hypoxia	Roots	Rocha et al. (2010)
Rice (*Oryza sativa*)		Hypoxia, nitrogen assimilation	Root	Reggiani et al. (1988)
Maize (*Zea mays*)	*alt*	Hypoxia, nitrogen stress	Roots	Muench et al. (1998)
Proso millet (*P. miliaceum)*	*AlaAT-2*	Nitrogen stress	–	Son et al. (1991, 1992)
Tomato (*Lycopersicon esculentum*)	*Solyc03g123610*	Hypoxia	Root	Czernicka et al. (2022)
Rice (*Oryza sativa*)	Os07g0617800	Yield under low Nitrogen	Plant	Rao et al. (2018)
Rice (*Oryza sativa*)	OsDIAT	Drought stress	Leaves and roots	Shim et al. (2023)
Rice (Oryza sativa)	AlaAT	High night temperature	Panicles	Schaarschmidt et al. (2020)
Rice (*Oryza sativa*)	OsAAP11 (Os11g0195600)	Grain quality, bud outgrowth and tillering	Endosperm	Yang et al. (2023); Wu et al. (2024)
Tobacco (*Nicotiana tabacum*)	co-AlaAT (a synthetic construct)	Nitrogen use efficiency	All parts of plants	Ahmed et al. (2020)

## Role of alanine aminotransferase in seed dormancy/pre-harvest sprouting tolerance

Preharvest sprouting tolerance is an important criterion for modern crop varieties in providing resilience against climate change. Rainfall during crop maturity stage makes the grain germinate prematurely and thus deteriorates in quality for consumption. The hypogeal crop like groundnut is very susceptible to preharvest sprouting during end season rains. Genes responsible for preharvest sprouting and/or dormancy were identified in major crops like wheat and barley. Such genes codes for alanine aminotransferase activity. We have emphasised here on detailed account of genetics experiment for mapping and cloning of such genes in barley and wheat and thereafter validation of the role of AlaAT in seed dormancy/preharvest sprouting tolerance.

### Identification of *AlaAT* gene for fresh seed dormancy in barley

Fresh seed dormancy rescues seeds from in situ germination in the matured plant or in the soil (for hypogeal fruits). Seed dormancy is widely desired and the most sought attribute to compensate the detrimental effect of unseasonal or end-seasonal rains due to climatic changes in cereals, pulses and oilseeds. Hence, induction and incorporation of seed dormancy in crop plants will be one of the prime strategies in addressing challenges arising from unseasonal rains. Infusion of seed dormancy for few weeks may save the seeds from germination due to unseasonal rains during harvest. The earliest reports on quantitative trait loci (QTL) identification for seed dormancy were published by Anderson et al. (1993) and Ullrich et al. (1992) in wheat and barley. Later, Kleinhofs et al. (1993) tagged QTLs determining seed dormancy (SD) in ‘Steptoe (dormant) x Morex (non-dormant)’ DH lines constructed by the North American barley genome mapping project. Of these few initial reports, four *SD* loci (*SD 1* to *SD 4*) were common, which were surrounded by designated RFLP markers and accounted for approximately 50, 15, 5 and 5% of the phenotypic variability due to seed dormancy, respectively (Ullrich et al. 1992; Oberthur et al. 1995). Han et al. (1996) analysed the progeny of different reciprocal crosses involving six double haploid (DH) lines derived from Steptoe (a dormant spring feed cultivar) and Morex (non-dormant) and revealed flanking markers Ale and ABC302 (for SD 1 locus) on the long arm of chromosome-07 (5H translocation). This *SD1* locus was found partly epistatic to the *SD2* locus that is present in between the interval of ABC309 and MWG851 markers near the telomere of the long arm of chromosome 07. In the absence of the *SD1* allele, *SD2* exerted moderate to large effects on dormancy. Larson et al. (1996) reported that the PSR128 marker locus on long arm of chromosome-7 (5H) accounted for a large portion of the phenotypic variation and had strong effects on germination percentage, especially when the Steptoe allele inhabited the second largest dormancy QTL near the ABG390 marker. The PSR128 marker explained 19.1% and 19.9% variation due to seed germination in Steptoe and Morex back-cross (BC) populations, respectively.

Later, Gao et al. (2003) mapped the SD2 locus to a 0.8 cM marker interval between MWG851D and MWG851B near the chromosome 7 (5H) telomere. Li et al. (2003) analyzed the QTL for seed dormancy and pre-harvest sprouting using a DH population derived from a cross of ‘Chebec x Harrington’ in barley and found one major QTL to control pre-harvest sprouting. This major QTL was located on chromosome 5HL and flanked by RFLP marker CDO506 and SSR marker GMS1. GMS1 marker was further validated in a ‘Stirling x Harrington’ DH population. Quantitative seed dormancy 1 (*qsd 1*) was identified as a single major dormancy QTL using DH lines derived from ‘HarunaNijo’ and the wild barley accession ‘H602’ (Hori et al. 2005, 2007a). HarunaNijo (malting barley) had 100% seed germination rates. By contrast, H602 is a wild progenitor of cultivated barley (*H. vulgare* ssp. *spontaneum*), and had very strong dormancy at harvest. Later, Sato et al. (2009) identified a major QTL in centromeric region of chromosome 5H (*Qsd1*) using the EST map based on ‘HarunaNijo (*H. vulgare* ssp. *vulgare*) x wild barley H602 (*H. vulgare* ssp. *spontaneum*)’ DH population and recombinant chromosome substitution lines. Sato et al. (2016) located *qsd1* in a 9,467 bp region between EST5 and EST4F (LC054176, AK372829) and found four single nucleotide polymorphisms (SNPs) in the coding region of AK372829 which encodes an AlaAT. Later, association mapping analysis of wild AlaAT genotypes and dormant phenotypes (mutant alaAT) suggested that a SNP G642C in the exon 9 of *AlaAT*, which could be a determinant nucleotide variation for dormancy in the studied genotypes. This SNP causes substitution of the 214th amino acid residue from leucine (L) in the dormant allele to phenylalanine (F) in the non-dormant allele in *AlaAT*.

Towards proof of function, knockdown plants were developed by RNA interference (RNAi) with the pANDA vector in Golden Promise variety (which carried the dominant short dormancy *Qsd1* allele) and found 5% germination in *qsd1/qsd1*, 40% germination in heterozygous plants and 80–90% germination in Golden Promise (Sato et al. 2016). On analysing the sequence, it was observed that qsd1 enzyme has a non-synonymous mutation at 214th amino acid residue, whereas phenylalanine (F) residue changed to leucine residue in wild barley H602. This seed dormancy gene was expressed specifically in the embryo. The AlaAT isoenzymes encoded by the long and short dormancy alleles differ in a single amino acid residue (L214F). It is believed that the reduced dormancy allele *Qsd1* evolved from wild barley that was first domesticated in the southern Levant and had the long dormancy *qsd1* allele that can be traced back to wild barley (Sato et al. 2016; Onishi et al. 2017). In contrast, the long dormancy allele might be used to control pre-harvest sprouting in higher rainfall areas to enhance global adaptation of barley. It will be interesting to look into the gene for seed dormancy/preharvest sprouting in major food crops like wheat. Apart from barley and wheat, dormancy in rice was reported to be involved in alanine production. A transcriptome study between dormant red rice and non-dormant rice revealed the possible involvement of phosphoenolpyruvate carboxykinase, pyruvate phosphate dikinase, and alanine aminotransferase pathways as an important gluconeogenetic pathway associated with the restoration of plastid functions in the dormant seed following imbibition (Gianinetti et al. 2018). While a mut-map study recently revealed a role of allantoate amidohydrolase (*OsAAH*) in controlling pre-harvest sprouting in a rice mutant. The disruption of *OsAAH* increased the level of ureides and activated the tricarboxylic acid cycle for energy production in germinating seeds to favour sprouting in the rice mutant (Xie et al. 2024). While in legume crops like common beans, the physiological mechanism towards seed dormancy was attributed to a non-functional pectin acetylesterase 8 (due to 5-bp insertion) possibly impairs the function of cell wall protrusion during radicle emergence (Soltani et al. 2021). On contrary, two candidate genes-*RING-H2 finger protein* and *zeaxanthin epoxidase* were responsible for seed dormancy in groundnut due to their role in seed development and abscisic acid accumulation (Kumar et al. 2020).

**Fig. 1:**
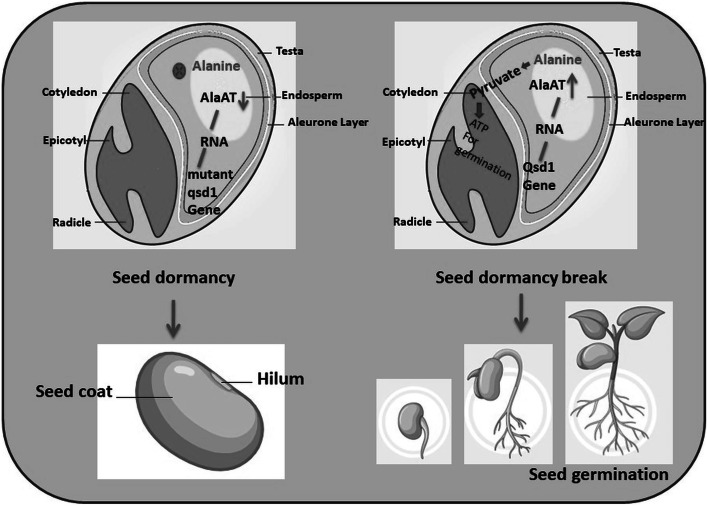
Schematic representation of the role of alanine aminotransferase in regulation of seed dormancy in barley and wheat. In case of dormancy (left panel), mutant *qsd1* gene do not produce functional alanine aminotransferase (AlaAT) and thus the reserve alanine not remobilize to produce energy through gluconeogenetic pathway. In contrary, the wild *QSD1* gene (right panel) produce functional AlaAT and converts reserve alanine to pyruvate for energy production during germination

During germination imbibed seeds produce energy to support processes essential for radicle protrusion (Rosental et al. 2014). Lehmann and Ratajczak (2008) suggested that stored amino acids can provide an alternative substrate for energy production during the first hours of imbibition. The level of ATP in imbibed seeds rises during the first 6 h (Botha et al. 1992). In general cereal seeds have a lower threshold of oxygen level needed to promote germination (Al-Ani et al. 1985). In such a situation fermentation plays a role that was exemplified by lactate abundance in Arabidopsis seeds following the first day after imbibition (Allen et al. 2010). During germination, ethanol fermentation increases in many seeds (Botha et al. 1992), and *alcohol dehydrogenase* transcripts are upregulated in barley and Arabidopsis seeds during early imbibition (Sreenivasulu et al. 2008; Weitbrecht et al. 2011). With this concept in mind, it is suggested that the non-dormant barley seeds are used to mobilize reserved alanine into pyruvate through the action of active alanine aminotransferase (product of *Qsd1*). While mutated *qsd1* can’t mobilize the alanine to pyruvate for energy production during the first hours of imbibition (Fig. 1).

### Translation of barley dormancy gene (*AlaAT*) information into wheat

Using a backcross reciprocal monosomic method, Miura et al. (2002) found that chromosome-3A and homologous group 04 chromosome were the possible sites of QTLs for the high level of seed dormancy in Zenkoujikomugi (Zen) wheat. Later, Mori et al. (2005) identified a major QTL responsible for grain dormancy in the proximal end of the short arm of chr-3A. This QTL (QPhs.ocs-3A.1) was mapped within the 4.6 cM region flanked by Xbarc310 and Xbcd907 with a phenotypic variation of 11.6–44.8%. Moreover, QPhs.ocs-4A.1 (the other QTL for dormancy) was identified on the long arm of chromosome 4A, and QPhs.ocs-4B.1, on the centromeric region of the long arm of chromosome 4B. Most of the transgressive segregants with higher dormancy among the 125 RILs had a combination of Zen wheat alleles at QPhs.ocs-3A.1 and QPhs.ocs-4A.1 and the Chinese Spring (CS) allele at QPhs.ocs-4B1 (Mori et al. 2005). The QTL QPhs.ocs-3A.1 was later identified to harbour MOTHER OF FT AND TFL1 (*MFT1*) that regulate the germination of wheat seed (Nakamura et al. 2011). The other wheat QTL on chromosome 4AL (*Phs-1*) was identified as an orthologue of barley *Qsd2* that encodes a MAP kinase (Torada et al. 2016). However, an ortholog of barley *Qsd1* (*ALT1*) was identified on chromosome 5 through a genetic mapping study in diploid einkorn wheat (*T. boeoticum* x *T. monococcum*) (Hori et al. 2007b). Later, Onishi et al. (2017) identified orthologs of barley *Qsd1* in hexaploid wheat and found three amino acid substitutions in the B sub-genome that may be associated with variation in the period of dormancy. Subsequent research validated the association of these orthologs of barley *Qsd1* with pre-harvest sprouting in wheat. Wei et al. (2019) isolated three barley *Qsd1* homologs in common wheat designated *TaQsd1-5A*, *TaQsd1-5B*, and *TaQsd1-5D*. Two cleaved amplified polymorphic sequence markers (QSD1 and QSD3) and one ‘Kompetitive Allele Specific PCR’ marker (QSD2) for *TaQsd1-5B* were developed and validated in a population consisting of 351 wheat varieties and a RIL population of ‘Yangxiaomai × Zhongyou 9507’. The marker Qsd1 co-segregated with a major QTL for the period of dormancy in the RIL population. The transcript levels of *TaQsd1-5B* were significantly higher in long-dormant lines than in short-dormant lines during seed imbibition, which is consistent with the activity of the AlaAT enzyme encoded by *TaQsd1-5B*. After the identification of *Qsd1* homologs in wheat (*TaQsd1*), site-directed mutagenesis was performed with *Agrobacterium*-mediated transformation with CRISPR/Cas9 gene cassette in wheat by Abe et al. (2019). Genome editing was attempted at the conserved region of exon 14 of *TaQsd1* in three sub-genomes. The triple-recessive mutant had a significantly longer germination period than other genotypes which indicated a frameshift of the nucleic acid sequence of *TaQsd1* at the 14th exon that caused a loss of function of *TaQsd1*, and altered the phenotype of the triple mutant. Liu et al. (2021) developed a method of *in planta* particle bombardment (iPB) to express CRISPR/Cas9 components intended to target *TaQsd1*, which were bombarded into the embryos of imbibed seeds with their shoot apical meristem (SAM) exposed. Genotypic analysis of single triple-recessive homozygous mutant T_2_ plants exhibited a 7 day delay in the time required for 50% seed germination compared to wild-type “Haruyokoi” (Liu et al. 2021). The iPB method was also successfully used in editing the genome of two elite winter cultivars, ‘Yumechikara’ and ‘Kitanokaori’, but efficiencies were lower as compared to ‘Haruyokoi’. Hisano et al. (2022) established Cas9-induced targeted mutagenesis to create T-DNA-free and homozygous *qsd1* mutants with a 1-bp insertion (*qsd1-1*), a 1-bp deletion (*qsd1-2*), or a 3-bp deletion (*qsd1-3*). While wild-type Qsd1 contained 494 amino acids, the protein was shortened by one amino acid in *qsd1-3*; the other mutants carried premature stop codons that led to truncated proteins of 72 (qsd1-1) amino acids. Prolonged dormancy in the *qsd1-3* allele mutant was observed which encoded a protein lacking, a histidine residue at position 32. This suggested that qsd1 might be a loss of function mutation in *Qsd1* and the deletion of a histidine residue may have affected the secondary structure of Qsd1, leading to an inactive protein. Huang et al. (2023) found a weak seed dormancy (WSD1), a new seed dormancy regulator in rice encoding an aminotransferase protein that regulates seed dormancy through the gibberellic acid (GA) biosynthesis pathway, abscisic acid signalling pathway and amino acid homeostasis. Through metabolome study, it was observed that GA1 content and expression of GA biosynthesis-related genes were increased in the *wsd1* mutant (higher seed germination) and reduced sensitivity to ABA compared with the wild-type (Huang et al. 2023).

### Phylogenetic relationship between the various alanine aminotransferases in seedling

**Fig. 2:**
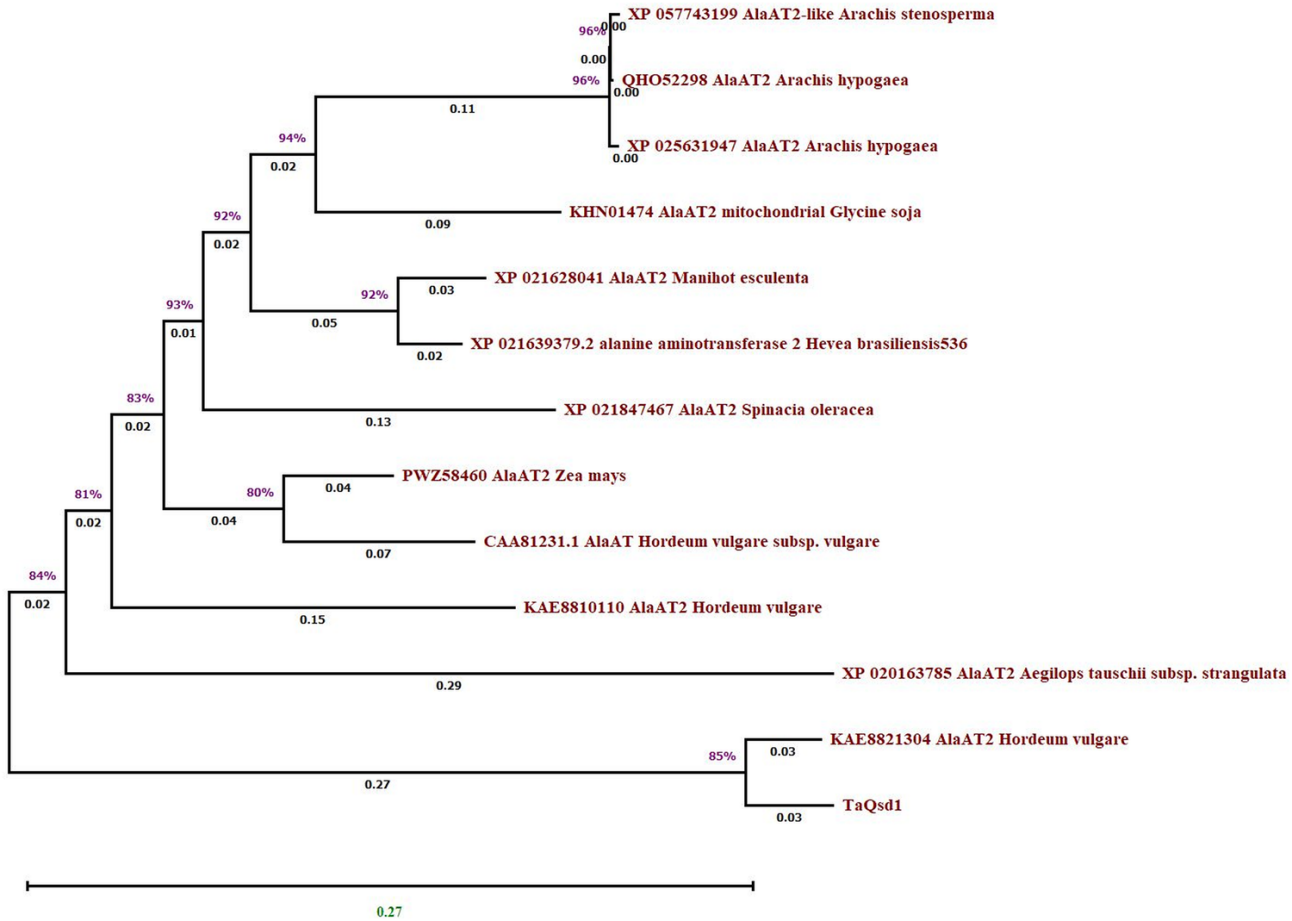
Phylogenetic relationship between the protein sequences of different plant species at seedling stage. The clear separation of monocot AlaAT and dicot AlaAT is depicted here. Monocot AlaAT includes KAE8821304.1 that codes for QSD1 gene in barley. Whereas the dicot AlaATs are group together and distantly placed from barley QSD1

It is very important to fish out seed tissue specific alanine aminotransferase gene from hypogeal crops like groundnut towards breeding for pre-harvest sprouting resistant varieties. Towards this attempt, we analysed the phylogenetic relationship among 13 different protein sequences of AlaAT from seed/seedling stage of different plant species including groundnut derived from a large set of AlaAT protein sequences available at NCBI (https://www.ncbi.nlm.nih.gov/) (Table 2). Alignment of these sequences was performed using MEGA11 through MUSCLE (Tamura et al. 2021). The evolutionary distances were computed using the Poisson correction method (Zuckerkandl and Pauling 1965) based on the units of the number of amino acid substitutions per site. The neighbor-joining phylogenetic tree that resulted from this analysis produced a clear separation into two subfamilies (Fig. 2), containing AlaAT2 from *Hordeum vulgare* (KAE8821304.1) responsible for seed dormancy in growing seedling and *Triticum aestivum Qsd1* (*TaQsd1*) together in subfamily B. Whereas other AlaAT2 from different plant species expressed in various tissues were clustered together in subfamily A. This indicates that the *TaQsd1* sequence present in *T. aestivum* is very similar in sequence with *H. vulgare* AlaAT2 codes for *Qsd1* gene responsible for seed dormancy in *H. vulgare*. The AlaAT2 from *Arachis* species (bears hypogeal fruits) showed more resemblance with mitochondrial AlaAT in soybean. DeRosa and Swick (1975) suggested that mitochondrial AlaAT is present in gluconeogenetic tissue that can utilize alanine for glucose production in animals. The configuration of alanine aminotransferase differed in monocot and eudicot species. C4 monocots recruited an AlaAT from a specific cytosolic branch, but eudicots use alanine AlaAT copies from a mitochondrial branch (Schlüter et al. 2019). Thus, care must be taken in selecting query sequence to find out different copies of AlaAT genes across groundnut genome.

**Table 2:** AlaAT expression in various tissues during seedling stage of different plant species

Plant species	Protein Id	Tissue expressed
*Arachis hypogaea*	QHO52298	Young leaf
*Arachis hypogaea*	XP_025631947	Etiolated young seedling
*Arachis stenosperma*	XP_057743199	Etiolated seedlings
*Glycine soja (mitochondrial)*	KHN01474.1	Root
*Spinaciaoleracea*	XP_021847467	Leaf
*Manihot esculenta*	XP_021628041	Leaf
*Zea mays*	PWZ58460.1	Seedling
*Hordeum vulgare*	KAE8810110.1	Whole seedling
*Aegilops tauschii subsp. Strangulate*	XP_020163785.1	Seedling
*Hordeum vulgare*	KAE8821304.1	Whole seedling
*Triticum aestivum*	TaQsd1	Seedling
*Hordeum vulgare subsp. vulgare*	CAA81231.1	Root
*Hevea brasiliensis*	XP_021639379.2	New sprout leaves
*Trifolium pratense*	PNY05134.1	Young leaves
*Panicum miliaceum*	CAA49199.1	Leaf tissue (greening stage)
*Trifolium repens*	KAK2427973.1	Leaf (matured plant stage)
*Salix purpurea*	KAJ6719623.1	Shoot tip
*Musa troglodytarum*	URE09954.1	Leaf (flowering stage)
*Vigna angularis*	KAG2380095.1	Leaf (squaring stage)
*Thalictrum thalictroides*	KAF5193609.1	Leaves (mature stage)
*Handroanthus impetiginosus*	PIN09984.1	Leaf (adult stage)
*Vitis vinifera*	XP_002265294.2	Leaf
*Malania oleifera*	XP_057979904.1	Leaf (mature stage)
*Magnolia sinica*	XP_058078403.1	Leaf
*Rhododendron vialii*	XP_058226929.1	Leaf (mature stage)
*Amaranthus tricolor*	XP_057548777.1	Leaf
*Actinidia eriantha*	XP_057460020.1	Leaf
*Syzygium oleosum*	XP_030468520.1	Lleaf (mature stage)
*Arachis duranensis*	XP_015948247.1	Whole plant
*Prosopis cineraria*	XP_054792497.1	Leaf
*Vigna angularis*	XP_017442520.1	Leaf (squaring stage)
*Lolium perenne*	XP_051191320.1	Leaf (established plant, before flowering)
*Cocos nucifera*	KAG1366389.1	Spear leaf of Hainan tall coconut
*Camellia sinensis*	AMR43300.1	Leaf
*Quillaja saponaria*	KAJ7953567.1	Leaf (cutting stage)
*Salix viminalis*	KAJ6715014.1	Shoot tip (mature stage)
*Raphanus sativus*	KAJ4879405.1	Leaf
*Euphorbia peplus*	WCJ39299.1	Young leaves/bracts (reproductive stage)
*Rhynchospora pubera*	KAJ4820413.1	Leaves
*Melia azedarach*	KAJ4711034.1	Leaf
*Hirschfeldia incana*	KAJ0257220.1	Leaf (full-grown, flowering stage)
*Triticum urartu*	XP_048567700.1	Leaf
*Ricinus communis*	XP_015583166.2	Leaves (Young leaves stage)
*Acacia crassicarpa*	LAG06236.1	Callus
*Capsicum annuum*	XP_016578512.1	Leaf
*Lolium rigidum*	XP_047045776.1	Leaves (mature plant stage)
*Citrus sinensis*	KAH9713885.1	Young leaves (mature stage)
*Erigeron canadensis*	XP_043623449.1	Leaves
*Zingiber officinale*	XP_042379321.1	Rhizome
*Eucalyptus grandis*	XP_010029516.2	Leaf (tree stage)
*Triticum dicoccoides*	XP_037453854.1	Leaf (vegetative stage)
*Senna tora*	KAF7813968.1	Leaf
*Helianthus annuus*	XP_021997041.1	Leaves (leaves stage)
*Tripterygium wilfordii*	KAF5743820.1	Leaf (mature plant stage)
*Vitis riparia*	XP_034696013.1	Leaf (Adult plant)
*Cannabis sativa*	XP_030490921.1	Leaves
*Nicotiana tomentosiformis*	XP_009606549.1	Leaf
*Carex littledalei*	KAF3339297.1	Leaf (anthesis stage)
*Cucumis sativus*	XP_004144444.2	Leaf
*Hibiscus syriacus*	KAE8707985.1	Leaf
*Malus domestica*	XP_008380735.1	Leaf (adult stage)
*Dendrobium catenatum*	XP_020704108.1	The whole plant (growing period stage)
*Panicum miliaceum*	RLN34837.1	Leaves
*Capsicum chinense*	PHU15002.1	Leaf, stem, root, flower, immature fruit, mature fruit
*Amaranthus tricolor*	ASL68820.1	Leaf
*Arachis ipaensis*	XP_016182704.1	Whole plant
*Amborella trichopoda*	XP_011628485.1	Leaf
*Dichanthelium oligosanthes*	OEL38824.1	Leaf (late vegetative developmental stage)

## Role of alanine aminotransferases in hypoxia

**Fig. 3:**
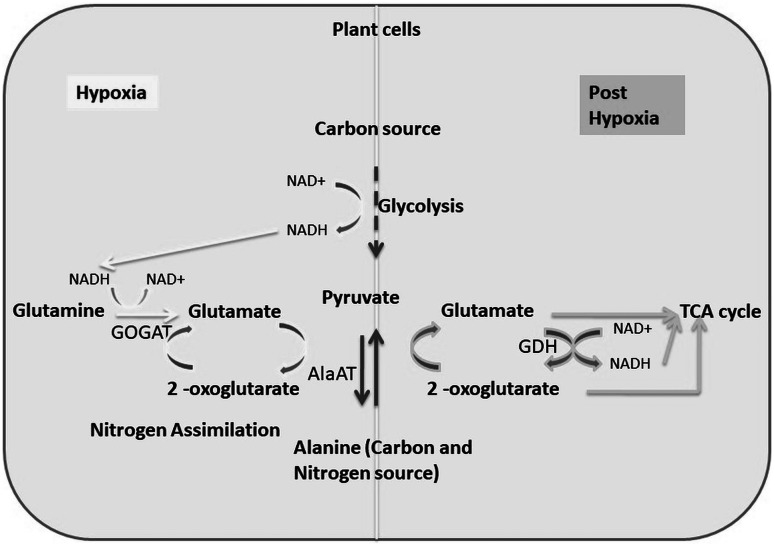
Representation of the conversion of pyruvate to alanine through an alternative pathway in hypoxia condition of plants. Under hypoxia (left panel) the pyruvate produced in glycolysis is converted to alanine as a nitrogen/carbon reserve through the mediation of GOGAT and AlaAT. The NADH produced in glycolysis is consumed in NADH-GOGAT pathway to glutamate which is ultimately utilized by AlaAT to form alanine and 2-oxoglutarate. While, in post hypoxia (right panel) situation, this reserve alanine was reused for pyruvate production through the mediation of AlaAT and GDH. The NADH produced in GDH pathway is used for energy production via oxidative phosphorylation

Hypoxia is a condition where oxygen supply decreases in plants and limits their energy production through the oxidative metabolic pathway. In hypoxia, an alternative pathway becomes active for ATP production. Under such conditions, the major sources of energy produced through glycolysis that generates 2 ATP and 2 pyruvate molecules with the reduction of NAD^+^ to NADH. To maintain glycolysis under anoxic conditions, NAD^+^ must be continuously regenerated from NADH via fermentative reactions. Using pyruvate as a substrate, fermentative metabolism either produces lactate via lactate dehydrogenase or ethanol via two subsequent reactions catalyzed by pyruvate decarboxylase and alcohol dehydrogenase (Tadege et al. 1999). However, these two pathways have clear drawbacks: lactate is toxic for the cells, and ethanol diffuses rapidly out of the cells, which leads to a considerable loss of carbon source during hypoxia. To minimize such loss of carbon, plants convert pyruvate to alanine through an alternative pathway as mentioned in Fig. 3, where the transamination reaction takes place through the action of alanine aminotransferase. The NADH thus produced during glycolysis is used in NADH-GOGAT pathways to re-generate NAD^+^ and convert glutamine to glutamate. Glutamate is reused by the AlaAT to form alanine and 2-oxoglutarate (Diab and Limami 2016). In support of such finding, Limami et al. (2008) demonstrated stimulated activity of NADH-GOGAT in response to hypoxia in *Medicago* sp.

De Sousa and Sodek (2003) studied the effect of hypoxia in the roots of soybean (*Glycine max* L. Merril cv. IAC-17) and found that alanine is one of the main products during anaerobic metabolism in plants and formed by AlaAT, an enzyme induced under such conditions. Similarly, many researchers analyzed that the AlaAT induced during hypoxia conditions in several plant species including *Hordeum vulgare* (barley) (Good and Crosby 1989; Muench and Good 1994) and *Zea mays* (maize) (Muench et al. 1998). The *AlaAT1* knockout mutant (alaat1-1) showed a dramatic reduction in AlaAT activity, suggesting that AlaAT1 is the major AlaAT isozyme in *Arabidopsis.* The reduction of AlaAT activity in the knock-out *Arabidopsis* was complemented through the over-expression of barley AlaAT (Miyashita et al. 2007). Further, up-regulation of AlaAT1 in *Arabidopsis* during hypoxia had also been demonstrated through microarray studies (Klok et al. 2002; Loreti et al. 2005). In *Arabidopsis*, four gene copies have the AlaAT activity. These are two *AlaATs* (*AlaAT1*, *At1g17290*; *AlaAT2*, *At1g72330*) and two glutamate-glyoxalate aminotransferases (*GGAT*) (*GGAT1, At1g23310; GGAT2, At1g70580*) (Igarashi et al. 2003; Liepman and Olsen 2003). Sequence identification revealed that Arabidopsis AlaAT1 and AlaAT2 have high similarity with barley (Muench and Good 1994), maize (Muench et al. 1998), and rice (Kikuchi et al. 1999). Strong expression of AlaAT1 was seen in both the roots and shoots, whereas weak expression of AlaAT2 was detected only in the shoots indicating that AlaAT1 is the major gene expressed in roots among the four members of the Arabidopsis AlaAT gene family (Miyashita et al. 2007). Moreover, Liepman and Olsen (2003) found that AlaAT1 is cytosolic and AlaAT2 is mitochondrial. Muench and Good (1994) cloned 1.75 kb cDNA and analyzed that the RNA blot analysis of barley root tissue showed a fourfold increase of a single AlaAT-2 mRNA band after 12—24 h of hypoxic stress, followed by a decrease in RNA levels after 48 h of hypoxic conditions. AlaAT-2 protein concentration increased in a similar pattern to AlaAT activity in root tissue, to almost sixfold the aerobic level after 96 h of hypoxic stress. AlaAT-2 activity increased more than twofold in roots of *Panicum miliaceum* exposed to hypoxia (Son et al. 1991; 1992). Kendziorek et al. (2012) supported the findings of four homologs of AlaAT in wheat where the activity of AlaAT1 in roots get induced in hypoxia condition than the other homologues AlaAT2, GGAT1 and GGAT2. They found a positive relation between the increment of AlaAT activity and alanine content in response to hypoxia conditions. The conversion of carbon source to alanine in roots during hypoxia is also mediated by the γ-aminobutyric acid (GABA) shunt pathway to a smaller extent. Miyashita and Good (2008) proved the above hypothesis by making two mutants defective in *glutamate decarboxylase* and *GABA transaminase* (*GABA-T*) wherein compromised alanine accumulation was noticed in response to hypoxia.

Under hypoxia, plants try to accumulate alanine to conserve both cellular carbon and nitrogen. During the reoxygenation after hypoxia, the alanine pool is used by the plants through the alanine aminotransferase/glutamate dehydrogenase cycle that yields pyruvate and NADH. Ultimately pyruvate and NADH are spent through the tricarboxylic acid (TCA) cycle during normoxic conditions. Cid et al. (2024) experimentally proved the important role of alanine as an amino-nitrogen donor and also a source for carbon skeletons to produce glucose de novo to meet the energy demand during waterlogging in wheat plants. Rocha et al. (2010) proved that the de novo nitrogen assimilation through nitrogen fixation is very important for survival of plants during hypoxia stress. Towards this, they have used transgenic *Lotus japonicas* having non-functional leghemoglobin gene (silenced by an RNAi approach) to study the contrasting behaviour of wild type and mutant under waterlogging condition. The mutant with non-functional leghemoglobin gene had reduced the root length in 1st week and underwent death of about 25% plants in 4 weeks period that demonstrated de novo nitrogen assimilation is an important criterion for survival of plant in waterlogging condition. Moreover, upregulation of glutamine synthetase and a subunit of nitrogenase complex (*nifH*) in the above mutant plants indicated nitrogen levels in the nodules were dropped. Drakeford et al. (1985) suggested a role of β-alanine in recovery from waterlogging resulted hypoxia in the plant in his hydroponic study. In Addition, some plants converted β-alanine to β-alanine betaine, a quaternary ammonium compound acts as an osmo-protectant that involved in tolerance to both salinity and hypoxia conditions (Hanson et al. 1991, 1994; van Dongen et al. 2009).

## Role of alanine aminotransferase in nitrogen-use efficiency

Nitrogen is one of the important macro-nutrients required by the plants for growth, development, reproduction and provides resistance against biotic and abiotic stresses. It is the main constituent of biochemical compounds that includes nucleic acids, amino acids, proteins, chlorophyll, and various secondary metabolites. Plants utilized inorganic (NO_3_^−^/NH_4_^+^) form of nitrogen through assimilation and metabolized using various enzymes. AlaAT is involved in nitrogen metabolism (Kishorekumar et al. 2020). The expression of AlaAT is regulated by light and nitrogen stress in the leaves of proso millet (*Panicum miliaceum*) (Son et al. 1991, 1992; Kendziorek et al. 2012). AlaAT was also reported to be up-regulated during recovery from nitrogen stress in maize (Muench et al. 1998). Genetically modified plants with overexpressed *AlaAT *from a barley *AlaAT* cDNA driven by a canola root-specific promoter (btg26) had increased biomass and seed yield compared to wild type under the condition of 40% lesser amount of applied nitrogen fertilizer required at field conditions (Good et al. 2007). Similarly, the transgenic rice plants with overexpression of barley *AlaAT* cDNA driven by a rice root tissue-specific promoter (*OsAnt1*) had high nitrogen use efficiency (Shrawat et al. 2008). In continuation with this higher nitrogen use efficiency in overexpressed transgenic plants, Beatty et al. (2009) studied the transcriptome in rice genotype overexpressing *AlaAT* and found that the *AlaAT* over-expression induced some of the genes that play a role in the nitrogen-use-efficient phenotype and related metabolite accumulation. Further, rice lines over-expressing barley alanine aminotransferase (*Hv*AlaAT), driven by the *OsAnt1* promoter, displayed higher root and shoot biomass production (Beatty et al. 2013; Selvaraj et al. 2017). Duff et al. (2012) showed that the AlaAT is involved in nitrogen assimilation, protein synthesis, and carbon metabolism. In the case of transgenic sugarcane and wheat plants with *OsAnt1::HvAlaAT* overexpression, improvements in nitrogen use efficiency and biomass production were noticed (Snyman et al. 2015; Peña et al. 2017). Rocha et al. (2010) studied the activity and gene expression of AlaAT in soybean roots and nodules and found that *GmAlaAT* responded to nitrogen availability in the solution during waterlogging. It was found from their study that the NH_4_^+^ form of nitrogen source induced both gene expression and enzyme activity of AlaAT more than when NO_3_^−^ was supplied in the nutrient solution. Sisharmini et al. (2019) successfully cloned the *AlaAT* gene from cucumber (*CsAlaAT2*) and overexpressed it in root tissue using the *OsAnt* promoter in rice to improve nitrogen use efficiency. Such finding demonstrate the importance of up-keeping of function of this gene in monocot as well as dicot for higher nitrogen use efficiency. In connection with these, a genome-wide association analysis in 472 rice genotypes revealed a significant association of alanine aminotransferase (*Os07g061780*) with the grain yield under low nitrogen (Rao et al. 2018; Jaiswal and Raghuram 2024). In addition, a ‘floury endosperm12’ mutant in rice represented a base substitution mutation (C to T in the 11th exon) in *OsAlaAT* gene, had loosely packed starch granules, lower amylose and higher protein content (Zhong et al. 2019). Overexpression of this gene in rice revealed concomitant up-regulation of genes involved in uptake and assimilation of nitrogen. Tiong et al. (2021) used genetically modified japonica rice (*Oryza sativa*, cv. Nipponbare) lines over-expressing *HvAlaAT* with *Os*Ant1 promoter. *OsAnt1::HvAlaAT* lines have increased above-ground biomass with little change to both nitrate and ammonium uptake rates under limited nitrogen. Further overexpression of the synthetic alanine aminotransferase (*co-AlaAT*) gene in tobacco revealed improvement in N uptake, total free amino acid, crude proteins, leaf area, stalk diameter, fresh weight of the plant, total dry matter, and seed weight per plant (Ahmed et al. 2020).

The use of mutant lines for nitrogen responsiveness has identified a mutant line with low nitrogen use efficiency 1 (lnue1*)* in the rice cultivar Zhongjiazao17. Subsequent map-based cloning of this lnue1 had identified a locus *LOC_Os10g25130* that codes an alanine aminotransferase (OsAlaAT1) in rice. Confirmation through gene-editing technique in wild-type plants had validated the similar phenotype in mutant plants. While overexpression of the isolated gene in a mutant background has increased the nitrogen use efficiency and starch deposition in grains and subsequent yield enhancement (Fang et al. 2022). This above example of identification of genes for nitrogen use efficiency in crop plants exemplified the importance of screening natural and inducing mutant populations for such agronomic traits and establishing the trait-function relationship. Recently, an environmental safety analysis of the effect of transgenic AlaAT overexpressing plants in soil rhizosphere was conducted by Yang et al. (2024). The overexpressing *pxAlaAT3* (*Populus xiaohei* AlaAT3) lines significantly increased the activity of acid phosphatase and protease under ammonium and nitrate conditions, respectively in soil rhizosphere. This increment of rhizosphere acid phosphatase activity is linked to more available phosphorus and plant vigour.

## Drought, salinity and heat stress tolerance trigger alanine aminotransferase

Drought and salinity limit crop yield and are becoming a major threat to global food security. Due to climate change, long-term drought spells, and rising sea levels are the limiting factors for the substantial expansion of agriculture in salinity and drought-affected areas. The uptake of nutrients and water is hindered by the salty soil and ultimately reduces crop productivity. Under the high salt condition, plants try to attempt cellular homeostasis by the production of various stress-associated endogenous metabolites that help to ease the stress condition. These metabolites significantly contribute to the survival and maintenance of growth and development of plants under salt stress (Zhao et al. 2020). Patel et al. (2020) and Zhou et al. (2024) revealed that plants undergo metabolic reprogramming that involves changes of primary and secondary metabolites to maintain appropriate osmotic homeostasis and activation of signaling pathways. These primary and secondary metabolites include alanine and β-alanine, respectively including other osmolytes. Rai and Sharma (1991) found that accumulation of β-alanine inhibits stomatal opening under drought conditions in *Vicia faba*. Further, the role of alanine in drought alleviation in *Phaseolus mungo* and Iris plants was reported (Rai 2002). β-alanine is produced through propionate which is sequentially converted through enzymatic catalysis to malonate semialdehyde. This malonate semialdehydes undergoes transamination by the mediation of L-alanine to β-alanine and pyruvate (Parthasarathy et al. 2019). β-alanine—α-alanine aminotransferases and β-alanine-pyruvate aminotransferases are involved in β-alanine metabolism in plants. The involvement of β-alanine aminotransferases via the production of β-alanine in alleviating drought and salinity stress in plants is depicted here. Three essential branched-chain amino acids amino acids (isoleucine, leucine, and valine) are accumulated copiously in plants in response to drought stress and act as compatible osmolytes or alternative energy sources (Joshi et al. 2010; Bowne et al. 2012; Fabregas and Fernie 2019). A direct evidence of the role of branched-chain amino acid aminotransferase in drought tolerance surfaced through overexpression of a cytoplasmic drought-induced branched-chain amino acid aminotransferase (*OsDIAT*) in rice where the overexpressed transgenic plant was found more tolerant to drought stress (Shim et al. 2023).

During exposure to soil salinity, plants have evolved various strategies to check the adverse effects of salinity, and several of them are connected to amino acid metabolism (Madhava Rao et al. 2006; Hildebrandt 2018). Such observation was made in cereal crops where alternation of amino acid abundance had decreased in tissues of barley (Wu et al. 2013), soybean (Lu et al. 2013), *Suaeda corniculate* (Pang et al. 2016), *Atriplex halimus* (Bendaly et al. 2016) and *Casuarina glauca* (Jorge et al. 2017). In contrast, Cao et al. (2017) reported that the concentrations of eight amino acids and amines increased significantly in barley under stress including 4-hydroxy-proline, asparagine, alanine, arginine, phenylalanine, citrulline, glutamine, and proline. Batista-Silva (2019) reported the upregulation of amino acid-derived secondary metabolites during the stress-recovery period in model plant Arabidopsis. Certain halophytic plants are known to accumulate quaternary ammonium compounds such as glycine betaine as an osmolyte in response to salt stress as an adjustment mechanism to protect their cells. A plant *Limonium vulgare* under Plumbaginaceae family is reported to accumulate β-alanine-betaine and choline-O-sulfate when grown in saline hypoxic conditions and they maintain osmotic balance without harming other physiological enzymes at higher concentrations inside the cell (Pollard and Wyn Jones 1979; Hanson et al. 1991, 1994). This quaternary osmoprotectant, β-alanine-betaine is synthesized from β-alanine (Hanson et al. 1994). In such a situation, it will be prudent to see the effect of spraying of β-alanine on seedlings in alleviating salt stress in plants. Ren and Chen (2023) studied the cotton seedlings to determine the role of β-alanine under 0.8% salt stress condition. Treatment with 25 mM β-alanine improved the photosynthetic efficiency due to higher expression of photosynthesis-antenna proteins and activation of hormones signal transduction pathways. Further transcriptome study revealed higher expression of various transcription factors, MYB (v-Myb myeloblastosis viral oncogene homolog), HD-ZIP (Homeodomain leucine zipper), ARF (Auxin response factors), MYC (Myelocytomatosis), EREB (Ethylene responsive element binding protein), DELLA (DELLA domain transcription factor), ABF (abscisic acid (ABA)-responsive element binding factor), H2A (Histone2A), H4 (Histone4), WRKY (WRKY domain transcription factor), and HK (Histidine kinases) involved in the regulation of salt tolerance and increased plant growth. β-alanine have also shown to improve the harmful effect of salt stress (decreasing of mitotic index, seed germination, seedling growth, chromosomal aberration) in *Allium cepa*. (Çavuşoğlu 2019). Both transcriptome and metabolome analyses in two contrasting sesame genotypes for salt tolerance revealed accumulation of more free amino acids including alanine, asparagine, aspartate, glutamate, isoleucine, leucine, valine etc. in tolerant than sensitive genotype (Zhang et al. 2019). While, transcriptome study detected significant higher changes in aspartate aminotransferase, branched-chain amino acid aminotransferase-2, glutamate decarboxylase in response to salt stress in this study.

Heat stress reduces the crop yield and its quality substantially. The negative effect of temperature rise due to climate change is predicted to decline around 6% in wheat production with each degree Celsius rise in temperature (Asseng et al. 2015; Kumar et al. 2023). The role of aminotransferase in counteracting heat stress is not known much. However, Arabidopsis plants showed a rapid increase in levels of sucrose and amino acids under high temperatures (Kaplan et al. 2004). These sucrose and amino acids derived from oxaloacetate and pyruvate through gluconeogenesis and alanine aminotransferase reaction, respectively in plants. Yamakawa and Hakata (2010) first time reported overexpression of alanine aminotransferase genes in response to high temperatures in developing grains of rice. Further, they opinioned that the accumulation of sucrose and amino acids is a general metabolic response to high temperatures in numerous plant species. The above finding was verified in a field experiment by Schaarschmidt et al. (2020) at IRRI, Philippines where they studied rice yield under high night temperatures as compared to daytime temperatures during the wet season and dry season. Metabolite composition analysis of such field experiment revealed significantly increased accumulation of alanine and 3-cyano alanine under ‘high night temperature’ conditions in panicles in the dry season, but not in the wet season.

## Role of aminotransferase in biotic stress resistance

Starvation of amino acid or amino acid-derived developmental regulators in plants may result in cross-talk in the defense pathway. Zhao et al. (1998) pointed out that amino acid starvation activates plant defenses. In living cells, aminotransferases are responsible for regulating the production of various amino acids. A direct role of aminotransferase in synthesizing antimicrobial compounds was evident from the action of lysine 2-aminotransferase in *Streptomyces virginae* in making cyclohexadepsipeptide antibiotic (Namwat et al. 2002). Evidence of the role of a plant aminotransferase against pathogen infection emerged from the analysis of aberrant growth and death2 (*agd2-1*) and AGD2-like defense response protein1 (*ald1*) mutants by Song et al. (2004). AGD2 and ALD1 are used to drive aminotransferase reactions in opposite directions and both enzymes have enzymatic activity with alanine, arginine, glutamine, methionine, leucine, and asparagine as amino donors. Further, ALD1 is used to upregulate upon pathogen infection in Arabidopsis. Song et al. (2004) postulated that possibly ALD1 synthesized a signal molecule important for inducing the synthesis of salicylic acid upon pathogen infection. The very role of ALD1 in disease resistance is further strengthened by the discovery of its biochemical activity in the production of pipecolic acid, a non-proteinogenic amino acid. ALD1 acts upon L-lysine and converts it through an aminotransferase reaction to ∆1-piperidine 2 carboxylic acid, which gets converted into pipecolic acid that mediates systemic acquired immunity in plants (Ding et al. 2016).

In a parallel finding, Taler et al. (2004) found that a gene encoding glyoxylate aminotransferases provided resistance against downy mildew in melon. This enzymatic resistance by glyoxylate aminotransferase is also correlated with higher levels of glycolate oxidase activity that catalyses the oxidation of glycolic acid to glyoxylate and hydrogen peroxide. The controlled overproduction of hydrogen peroxide at the infection site may impart an important signalling role in the induction of hypersensitive response towards providing resistance to downy mildew (*Pseudoperonospora cubensis*) in the melon leaf. The involvement of alanine aminotransferase (EC 2.6.1.2) in providing resistance against tobacco mosaic virus in hot pepper (*Capsicum annum* L. cv. Bugang) was first narrated by Kim et al. (2005). Expression of this *AlaAT1* gene in *Capsicum annum* is markedly induced in incompatible disease reaction as well as in response to the treatment of salicylic acid and jasmonic acid. During viral infection in leaf, the elevated demand of ATP in the infected tissue is fulfilled by the reserve alanine. In this scenario, AlaAT1 was used to convert reserved alanine into pyruvate in the cytosol and the pyruvate was spent for energy production (Kim et al. 2005). Towards the infestation of sucking insect pests in wheat and barley, a strong systemic increase in essential amino acids was evident in the past (Sandström et al. 2000). Further, a similar response in amino acid increase is altered in the metabolic pools of relatively resistant cultivars (Sempruch and Ciepiela 2001). This fluctuation of the amino acids pool is mainly driven by the activity of aminotransferase enzymes in plants. Towards its direct biochemical evidence, Sempruch et al. (2012) found an increase in AlaAT activity and the concomitant decrease in AspAT activity in aphid-infested aerial parts of triticale. Recent studies have identified a branched-chain aminotransferase (BCAT1) as a susceptibility factor for wheat leaf and stem rust. Knockout mutants of *TaBCAT1* have increased levels of branched-chain amino acids and enhanced levels of salicylic acid-dependent systemic acquired resistance response (Corredor-Moreno et al. 2021). A similar target for soybean cyst nematode pathogen (*Heterodera glycines*) effector, cysteine protease-1 was unveiled in soybean by Margets et al. (2024). Such a type of branched-chain aminotransferase can be engineered to provide resistance against disease-causing pathogens.

## Conclusion and future perspectives

Alanine aminotransferase is a vital biological enzyme that is found universally in all living organisms. While its functions vary between plants and animals, AlaAT plays significant role in ensuring the survival and lifespan of these organisms in certain limiting conditions. Understanding the concepts related to AlaAT, the genes responsible for its synthesis, and its diverse roles in plant functions are crucial for addressing agricultural challenges. In the context of climate change, a looming global challenge, comprehending the genetics of traits that enable survival and adaptation becomes imperative. AlaAT plays a key role in stress situations by assimilating metabolites and enhancing nutrient uptake, which is particularly vital for plants. Efficient nitrogen usage by plants is crucial, as it reduces the need for excessive nitrogen fertilizers, thereby minimizing negative environmental impacts due to eutrophication. One of the noteworthy contributions of AlaAT in plants is its involvement in seed dormancy. Seed dormancy is influenced by climate change, impacting agriculture significantly. Hypogeal crop plants like groundnut faces challenges due to untimely rainfall in semi-arid tropical regions. Towards these, it is important to harness the natural allele diversity in AlaAT gene over the global crop germplasm, in particular in hypogeal crop like groundnut. Availability of genome sequence information in groundnut accessions (https://www.peanutbase.org/) will help to reveal different orthologous and paralogous genes in this crop. Further, it is possible to generate SSR and SNP markers around the genomic regions of this ortholog/paralog of AlaAT. Such newly designed SSR and SNP markers will help to reveal trait based genetic variability for this target gene and assist in mining useful alleles for incorporation of the dormant trait in elite varieties. The recombination breeding approach can be integrated with rapid generation advancement protocol/speed breeding to quickly infuse the useful dormant allele into elite lines.

**Fig. 4:**
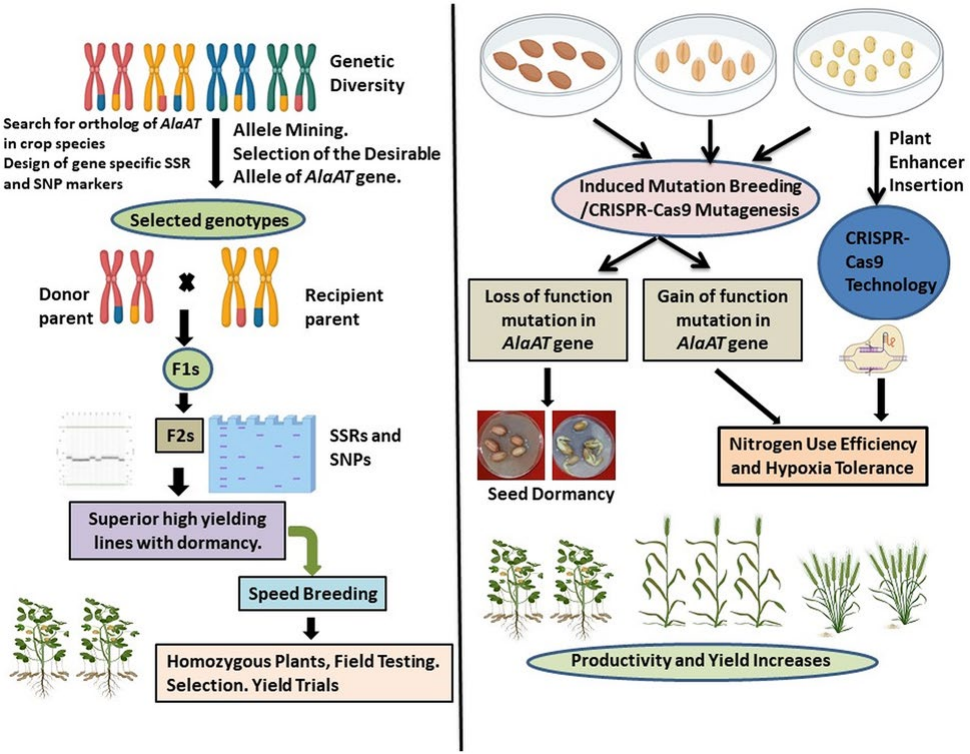
Future direction towards improvement of plant traits through manipulation of *AlaAT*. Importance of AlaAT and their homologs is narrated in this review article in respect to barley, wheat, rice. For a hypogeal crop (like groundnut), mining of AlaAT homologs (as a query sequence from barley/wheat) and detection of allelic diversity for the trait of interest can be undertaken from the available germplasm of the crop. This allelic diversity can be assessed by designing SSR markers around the AlaAT genomic loci or development of SNP markers within the gene itself. Once, the marker-trait association established in the available germplasm, the trait (for example, seed dormancy or nitrogen use efficiency) can be introgressed into high yielding varieties through marker-assisted selection. The isolated genotype (homozygous for the interested locus) will be rapidly fixed for the other background genome by speed breeding technique for field testing (left panel). Alternatively, *AlaAT* gene variability can be created through induced mutagenesis (by applying TILLING approach) or genome editing (CRISPR-Cas9). The loss of function of *AlaAT* can be exploited for isolation of dormancy genotype. While, gain of function mutants (can also be generated through enhancer insertion) will be of valuable material towards isolation of nitrogen use efficient and/or hypoxia tolerant genotypes (right panel)

Since the loss of function, the allele of AlaAT is providing dormancy in the case of barley and wheat; induced mutation breeding can be a viable approach to induce such a trait in a crop where natural trait diversity is almost meager. Further, induced mutation breeding will be an important tool to introduce loss of function allele in those crops that are not amenable to tissue culture and genetic engineering protocols (Fig. 4). AlaAT plays a significant role in alleviating the hypoxia stress. Short submergence exposure in many arable crops can be fatal due to a reduction in overall energy metabolism. Tissue-specific transient (hypoxia-related) overexpression of this gene may lead to tolerance against such a short period of submergence (Fig. 4). Towards the development of hypoxia-tolerant genotypes, root tissue-specific promoter (*OsAnt1* promoter), submergence-specific promoter (Mohanty 2021) and *AtERF71*/*HRE2* (Seok et al. 2022) will be helpful for transient expression in tissue specific manner. Recent findings from Claeys et al. (2024) have documented improvement in AlaAT gene expression in maize by inserting precise Plant Enhancer (PE) elements thereby improving nitrogen use efficiency. This PE element is 12 bp sequences. Insertion of different palindromic PE in the targeted promoter of AlaAT can be achieved through CRISPR-Cas9 technology towards the improvement of nitrogen use efficiency in plants (Fig. 4). Apart from the AlaAT, the branched-chain aminotransferase which acts as a susceptibility factor for disease development will be an important target for targeted mutagenesis. Such aminotransferase will be targeted in the future to generate variants through genome editing tools to develop disease-tolerant/resistant genotypes. Apart from the gene expression regulation, the stress response may change the protein activity through PTMs. The changes of protein and their activity can directly be detected through proteomics or indirectly through metabolomics. A typical metabolomic profiling is helpful to ascertain the role of certain metabolites. While, an isotope-labelled precursor can be introduced into plant tissues and redistribution of this precursor and downstream metabolites can be detected through Mass Spectrometry (MS) or Nuclear Magnetic resonance (NMR). Further, Mass Spectrometry Imaging (MSI) offers greater capability to simultaneously capture the spatial distribution of metabolites and macromolecules at the molecular level. With these things in mind, alanine (or other substrates of AlaAT) can be isotopically labelled and its fate can be checked through MS, NMR and/or MSI. Future research towards this direction may generate enough clues towards the role of alanine aminotransferase in various stress in a time and tissue specific manner. This discussion underscores the major role of AlaAT in plants, highlighting its contributions to seed dormancy, nitrogen use efficiency, and adaptation to hypoxic conditions. While much is known, numerous questions remain unanswered regarding other functions and climatic triggers in plants. Advancing the field of plant science is imperative, and maintaining an open mind is essential to uncover exciting possibilities within biological systems.

## Data Availability

All the data analyzed during this study are included in this review article. Data sharing is not applicable to this article as no new data was created.
